# Crystal structure of 2,5-bis­(di­phenyl­phosphan­yl)furan

**DOI:** 10.1107/S2056989015020964

**Published:** 2015-11-11

**Authors:** Carla Martínez de León, Hugo Tlahuext, Jean-Michel Grévy

**Affiliations:** a Centro de Investigaciones Químicas, Universidad Autónoma del Estado de Morelos, Av. Universidad 1001 Col. Chamilpa, CP 62209, Cuernavaca Mor., Mexico

**Keywords:** crystal structure, bis­(di­phenyl­phosphan­yl)furan, metal complexes, diphosphine ligands for catalysis, C—H⋯π inter­actions.

## Abstract

In the title compound, C_28_H_22_OP_2_, each of the P atoms has an almost perfect pyramidal geometry, with C—P—C angles varying from 100.63 (10) to 102.65 (9)°. In the crystal, neighbouring mol­ecules are linked *via* weak C—H⋯π inter­actions, forming supra­molecular chains along the *b*-axis direction.

## Related literature   

For the uses of rigid diphosphine compounds in the preparation of homo- or hetero-bimetallic complexes, which have high potential for specific applications in catalytic processes, see: Kaeser *et al.* (2013 ▸); Xu *et al.* (2014 ▸). For the structural characteristics of these ligands providing control over the distance separating the two metallic centers and consequently, over the properties of the corresponding complexes, see: Brown & Lucy (1986 ▸). For the synthesis of bis­(di­phenyl­phosphan­yl)furan, see: Brown & Canning (1983 ▸). For the resulting bimetallic complexes with Rh and Ir, see: Brown *et al.* (1984 ▸). For C—H⋯π inter­actions, see: Munshi & Guru Row (2005 ▸).
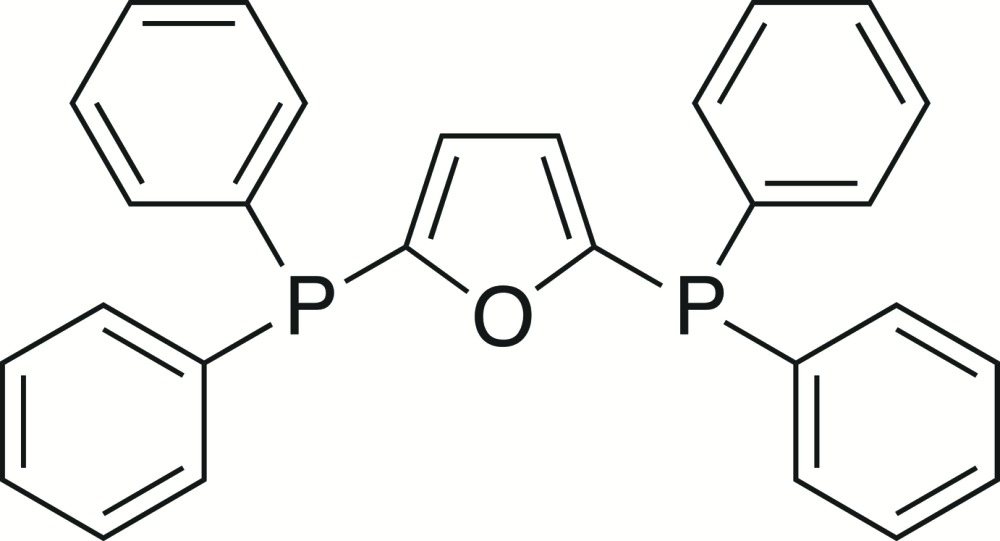


## Experimental   

### Crystal data  

C_28_H_22_OP_2_
*M* *_r_* = 436.40
Monoclinic, 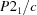

*a* = 10.7179 (9) Å
*b* = 8.5559 (7) Å
*c* = 24.550 (2) Å
β = 94.309 (1)°
*V* = 2244.9 (3) Å^3^
*Z* = 4
Mo *K*α radiation
μ = 0.21 mm^−1^
*T* = 100 K
0.17 × 0.15 × 0.12 mm

### Data collection  

Bruker SMART APEX CCD area-detector diffractometer
Absorption correction: multi-scan (*SADABS*; Bruker, 2000 ▸) *T*_min_ = 0.965, *T*_max_ = 0.975
17894 measured reflections
3952 independent reflections
3836 reflections with *I* > 2σ(*I*)
*R*_int_ = 0.045

### Refinement  

*R*[*F* ^2^ > 2σ(*F* ^2^)] = 0.046
*wR*(*F* ^2^) = 0.106
*S* = 1.17
3952 reflections
280 parameters
H-atom parameters constrained
Δρ_max_ = 0.49 e Å^−3^
Δρ_min_ = −0.24 e Å^−3^

### 

Data collection: *SMART* (Bruker, 2000 ▸); cell refinement: *SAINT* (Bruker, 2000 ▸); data reduction: *SAINT*; program(s) used to solve structure: *SHELXS97* (Sheldrick, 2008 ▸); program(s) used to refine structure: *SHELXL97* (Sheldrick, 2008 ▸); molecular graphics: *SHELXTL* (Sheldrick, 2008 ▸); software used to prepare material for publication: *SHELXTL*, *PLATON* (Spek, 2009 ▸) and *publCIF* (Westrip, 2010 ▸).

## Supplementary Material

Crystal structure: contains datablock(s) I. DOI: 10.1107/S2056989015020964/su5229sup1.cif

Structure factors: contains datablock(s) I. DOI: 10.1107/S2056989015020964/su5229Isup2.hkl

Supporting information file. DOI: 10.1107/S2056989015020964/su5229Isup3.cml

**Fig. 1.**
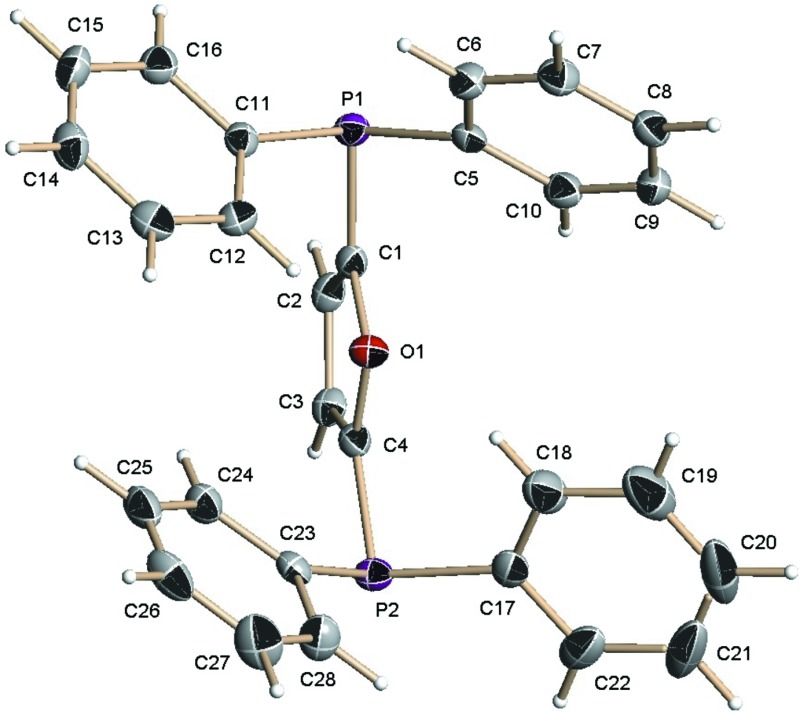
The molecular structure of the title compound, with atom labeling. Displacement ellipsoids are drawn at the 50% probability level.

. DOI: 10.1107/S2056989015020964/su5229fig1.tif
The mol­ecular structure of the title compound, with atom labeling. Displacement ellipsoids are drawn at the 50% probability level.

**Fig. 2.**
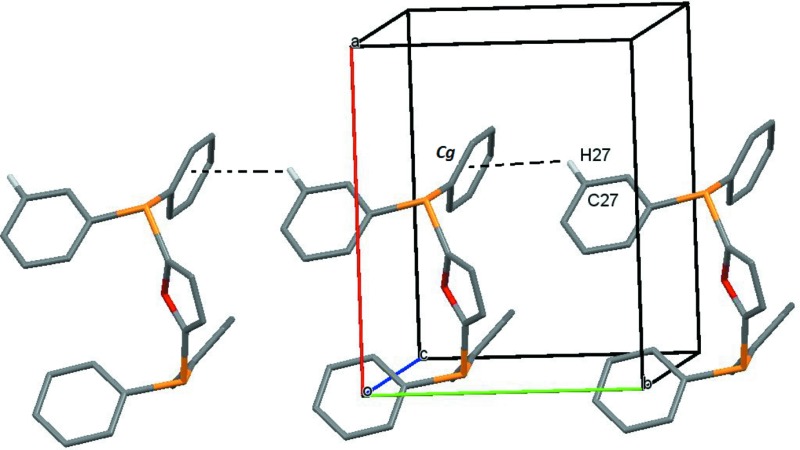
View of the C—H··· π interactions (dashed lines; see Table 1) linking adjacent molecules. Hydrogen atoms not involved in these interactions have been omitted for clarity.

. DOI: 10.1107/S2056989015020964/su5229fig2.tif
View of the C—H⋯ π inter­actions (dashed lines; see Table 1) linking adjacent mol­ecules. Hydrogen atoms not involved in these inter­actions have been omitted for clarity.

CCDC reference: 1435225

Additional supporting information:  crystallographic information; 3D view; checkCIF report

## Figures and Tables

**Table 1:** Hydrogen-bond geometry (Å, °) *Cg* is the centroid of ring C17–C22.

*D*—H⋯*A*	*D*—H	H⋯*A*	*D*⋯*A*	*D*—H⋯*A*
C27—H27⋯*Cg* ^i^	0.95	3.11	3.736 (3)	125

Symmetry code: (i) 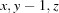.

## supplementary crystallographic information

### S1. Commentary

Rigid diphosphine compounds are important ligands for inorganic chemists as they can be used in the preparation of homo- or hetero-bimetallic complexes, which have high potential for specific applications in catalytic processes (Kaeser *et al.*, 2013; Xu *et al.*, 2014). The structural characteristics of these ligands provide control, among other things, over the distance separating the two metallic centers and consequently, over the properties of the corresponding complexes (Brown *et al.*, 1986). Thus, as part of an investigation in the field, some thirty years ago (Brown *et al.*, 1983) bis­(di­phenyl­phosphanyl)furan was synthesized for selective binuclear chelation and the resulting bimetallic complexes with Rh and Ir were isolated and latter tested in alkene hydrogenation (Brown *et al.*, 1984), showing a poorer activity than the corresponding mononuclear analogues. However, we believe that this diphosphine ligand is still of great inter­est for an exhaustive coordination study. In former reports the ligand was not spectroscopically characterized, nor its crystal structure determined, so here we report its full characterization and solid-state structure studied by single-crystal X-ray diffraction.

The molecular structure of the title compound, Fig. 1, shows the two phospho­rus atoms, P1 and P2, with almost perfect pyramidal geometry; the C—P—C angles are in a range of 100.63 (10) to 102.65 (9)°. The phenyl rings (C5—C10, C11—C16, C17—C22, C23—C28) and the furanyl ring (C1—C4/O1) are almost planar with r.m.s. deviations of 0.0024, 0.0019, 0.0026, 0.0072 and 0.0047 Å, respectively. The bond distances and angles have normal values.

In the crystal, the packing is stabilized via weak C—H···π inter­actions (Munshi & Guru Row, 2005), involving adjacent molecules, forming a supra­molecular chain along the *b* axis direction (Table 1 and Fig. 2).

### S2. Synthesis and crystallization

Although the title compound could be prepared in high yields by reaction between dili­thio­furan and 2 equivalents of chloro­diphenyl­phosphine (Brown & Canning, 1983), here it was obtained in 23% yield as a side product from the synthesis of 2-(di­phenyl­phosphanyl)furan: nBuLi in hexane solution (8.25 mmol) was slowly added to a furane solution (8.25 mmol) in 15 ml of Et_2_O. After two hours of stirring at room temperature, a 15 ml benzene solution of 1 equivalent of Ph_2_PCl was added drop wise at 273 K. After stirring the mixture overnight, all volatiles were eliminated under reduced pressure, and the resulting oil was diluted in CH_2_Cl_2_ and then filtered over Celite. The pure diphosphine was obtained as the second product eluted on a silica column with the solvent system Hexane: CH_2_Cl_2_ (80:20). Yield: 23%; m.p. 421 K; MS (FAB+) 436 m/z (M+) 40%; ^31^P NMR (CDCl_3_, 80 MHz, 20°C) -27.4 p.p.m; ^1^H NMR (400 MHz, CDCl_3_, 20°C): δ = 6.74 (m, 2H), 7.23-7.36 (m, 20H); RMN^13^C (100 MHz, CDCl_3_, 20°C); 122.62 (dd, 2C, ^2^*J*_CP_ = 27.8 Hz, ^3^*J*_CP_ = 7.3 Hz), 133.39 (d, 8C, ^3^*J*_CP_ = 19 Hz), 128.3 (d, 8C, ^3^*J*_CP_ = 7.3 Hz), 128.7 (s, 4C), 157.8 (d, 2C, ^1^*J*_CP_ = 24.9 Hz), 136.0 (d, 4C, ^1^*J*_CP_ = 4.4 Hz). Single crystals suitable for X-ray diffraction were grown by slow evaporation of a di­chloro­methane solution of the title compound at room temperature.

### S3. Refinement

Crystal data, data collection and structure refinement details are summarized in Table 2. H atoms were positioned geometrically and constrained using the riding-model approximation: *C*-H_phenyl_ = 0.95 Å with *U*_iso_(H_phenyl_)= 1.2 *U*_eq_(C), and *C*-H_furanyl_ = 0.95 Å, with *U*_iso_(H_furanyl_) = 1.2 *U*_eq_(C).

### Crystal data

**Table:**

C_28_H_22_OP_2_	*F*(000) = 912
*M**_r_* = 436.40	*D*_x_ = 1.291 Mg m^−^^3^
Monoclinic, *P*2_1_/*c*	Mo *K*α radiation, λ = 0.71073 Å
Hall symbol: -P 2ybc	Cell parameters from 7667 reflections
*a* = 10.7179 (9) Å	θ = 2.4–28.3°
*b* = 8.5559 (7) Å	µ = 0.21 mm^−^^1^
*c* = 24.550 (2) Å	*T* = 100 K
β = 94.309 (1)°	Block, colorless
*V* = 2244.9 (3) Å^3^	0.17 × 0.15 × 0.12 mm
*Z* = 4	

### Data collection

**Table:**

Bruker SMART APEX CCD area-detector diffractometer	3952 independent reflections
Radiation source: fine-focus sealed tube	3836 reflections with *I* > 2σ(*I*)
Graphite monochromator	*R*_int_ = 0.045
Detector resolution: 8.3 pixels mm^-1^	θ_max_ = 25.0°, θ_min_ = 1.9°
phi and ω scans	*h* = −12→12
Absorption correction: multi-scan (*SADABS*; Bruker, 2000)	*k* = −9→10
*T*_min_ = 0.965, *T*_max_ = 0.975	*l* = −29→29
17894 measured reflections	

### Refinement

**Table:**

Refinement on *F*^2^	Primary atom site location: structure-invariant direct methods
Least-squares matrix: full	Secondary atom site location: difference Fourier map
*R*[*F*^2^ > 2σ(*F*^2^)] = 0.046	Hydrogen site location: inferred from neighbouring sites
*wR*(*F*^2^) = 0.106	H-atom parameters constrained
*S* = 1.17	*w* = 1/[σ^2^(*F*_o_^2^) + (0.0276*P*)^2^ + 1.9895*P*] where *P* = (*F*_o_^2^ + 2*F*_c_^2^)/3
3952 reflections	(Δ/σ)_max_ = 0.001
280 parameters	Δρ_max_ = 0.49 e Å^−^^3^
0 restraints	Δρ_min_ = −0.24 e Å^−^^3^

### Special details

**Table:**

Geometry. All e.s.d.'s (except the e.s.d. in the dihedral angle between two l.s. planes) are estimated using the full covariance matrix. The cell e.s.d.'s are taken into account individually in the estimation of e.s.d.'s in distances, angles and torsion angles; correlations between e.s.d.'s in cell parameters are only used when they are defined by crystal symmetry. An approximate (isotropic) treatment of cell e.s.d.'s is used for estimating e.s.d.'s involving l.s. planes.
Refinement. Refinement of *F*^2^ against ALL reflections. The weighted *R*-factor *wR* and goodness of fit *S* are based on *F*^2^, conventional *R*-factors *R* are based on *F*, with *F* set to zero for negative *F*^2^. The threshold expression of *F*^2^ > σ(*F*^2^) is used only for calculating *R*-factors(gt) *etc*. and is not relevant to the choice of reflections for refinement. *R*-factors based on *F*^2^ are statistically about twice as large as those based on *F*, and *R*- factors based on ALL data will be even larger.

### Fractional atomic coordinates and isotropic or equivalent isotropic displacement parameters (Å^2^)

**Table:**

	*x*	*y*	*z*	*U*_iso_*/*U*_eq_	
C1	0.19328 (18)	0.3613 (2)	0.06380 (8)	0.0188 (4)	
C2	0.22310 (19)	0.4340 (2)	0.01764 (8)	0.0209 (4)	
H2	0.1689	0.4962	−0.0058	0.025*	
C3	0.35086 (19)	0.3998 (2)	0.01087 (8)	0.0213 (4)	
H3	0.3976	0.4334	−0.0184	0.026*	
C4	0.39337 (18)	0.3108 (2)	0.05365 (8)	0.0191 (4)	
C5	0.08909 (18)	0.3614 (2)	0.16627 (8)	0.0205 (4)	
C6	0.0223 (2)	0.2789 (3)	0.20342 (9)	0.0247 (5)	
H6	−0.0398	0.2060	0.1904	0.030*	
C7	0.0456 (2)	0.3020 (3)	0.25921 (9)	0.0270 (5)	
H7	−0.0005	0.2448	0.2841	0.032*	
C8	0.1353 (2)	0.4078 (3)	0.27864 (9)	0.0270 (5)	
H8	0.1505	0.4242	0.3168	0.032*	
C9	0.2031 (2)	0.4898 (3)	0.24222 (9)	0.0284 (5)	
H9	0.2658	0.5617	0.2555	0.034*	
C10	0.18003 (19)	0.4675 (3)	0.18634 (9)	0.0249 (5)	
H10	0.2265	0.5249	0.1616	0.030*	
C11	0.01519 (19)	0.1311 (2)	0.08714 (8)	0.0202 (4)	
C12	0.09903 (19)	0.0193 (3)	0.10970 (9)	0.0234 (5)	
H12	0.1751	0.0516	0.1288	0.028*	
C13	0.0715 (2)	−0.1378 (3)	0.10433 (9)	0.0270 (5)	
H13	0.1291	−0.2131	0.1197	0.032*	
C14	−0.0391 (2)	−0.1867 (3)	0.07683 (9)	0.0292 (5)	
H14	−0.0577	−0.2950	0.0736	0.035*	
C15	−0.1225 (2)	−0.0770 (3)	0.05413 (9)	0.0303 (5)	
H15	−0.1984	−0.1100	0.0351	0.036*	
C16	−0.0955 (2)	0.0810 (3)	0.05923 (9)	0.0248 (5)	
H16	−0.1530	0.1558	0.0435	0.030*	
C17	0.5853 (2)	0.3097 (2)	0.14057 (9)	0.0252 (5)	
C18	0.5087 (2)	0.2928 (3)	0.18342 (10)	0.0378 (6)	
H18	0.4317	0.2381	0.1777	0.045*	
C19	0.5435 (3)	0.3546 (4)	0.23426 (12)	0.0595 (9)	
H19	0.4902	0.3431	0.2632	0.071*	
C20	0.6558 (3)	0.4332 (4)	0.24305 (15)	0.0698 (12)	
H20	0.6795	0.4757	0.2781	0.084*	
C21	0.7333 (3)	0.4501 (3)	0.20137 (15)	0.0592 (10)	
H21	0.8108	0.5034	0.2077	0.071*	
C22	0.6986 (2)	0.3893 (3)	0.15011 (12)	0.0390 (6)	
H22	0.7521	0.4019	0.1213	0.047*	
C23	0.51056 (19)	0.0292 (2)	0.08703 (8)	0.0205 (4)	
C24	0.4083 (2)	−0.0493 (3)	0.06230 (9)	0.0264 (5)	
H24	0.3509	0.0047	0.0376	0.032*	
C25	0.3891 (2)	−0.2063 (3)	0.07329 (10)	0.0316 (5)	
H25	0.3185	−0.2588	0.0562	0.038*	
C26	0.4717 (2)	−0.2864 (3)	0.10876 (10)	0.0349 (6)	
H26	0.4575	−0.3932	0.1168	0.042*	
C27	0.5758 (3)	−0.2098 (3)	0.13253 (11)	0.0405 (6)	
H27	0.6346	−0.2650	0.1562	0.049*	
C28	0.5942 (2)	−0.0540 (3)	0.12202 (10)	0.0315 (5)	
H28	0.6653	−0.0022	0.1390	0.038*	
O1	0.29720 (12)	0.28470 (16)	0.08728 (6)	0.0202 (3)	
P1	0.04394 (5)	0.34210 (6)	0.09286 (2)	0.02035 (15)	
P2	0.54811 (5)	0.23216 (6)	0.07140 (2)	0.02108 (15)	

### Atomic displacement parameters (Å^2^)

**Table:**

	*U*^11^	*U*^22^	*U*^33^	*U*^12^	*U*^13^	*U*^23^
C1	0.0193 (10)	0.0148 (10)	0.0221 (10)	0.0016 (8)	−0.0010 (8)	−0.0015 (8)
C2	0.0247 (11)	0.0151 (10)	0.0223 (11)	−0.0027 (8)	−0.0027 (8)	0.0022 (8)
C3	0.0237 (10)	0.0190 (11)	0.0213 (11)	−0.0057 (8)	0.0022 (8)	0.0015 (8)
C4	0.0197 (10)	0.0165 (10)	0.0215 (10)	−0.0032 (8)	0.0041 (8)	−0.0015 (8)
C5	0.0174 (10)	0.0204 (11)	0.0238 (11)	0.0051 (8)	0.0017 (8)	−0.0031 (9)
C6	0.0223 (11)	0.0248 (12)	0.0270 (11)	−0.0023 (9)	0.0018 (9)	−0.0008 (9)
C7	0.0258 (11)	0.0301 (13)	0.0257 (12)	0.0011 (9)	0.0054 (9)	0.0008 (9)
C8	0.0251 (11)	0.0324 (13)	0.0233 (11)	0.0058 (10)	0.0002 (9)	−0.0058 (9)
C9	0.0235 (11)	0.0268 (12)	0.0345 (13)	−0.0017 (9)	0.0006 (9)	−0.0091 (10)
C10	0.0225 (11)	0.0214 (11)	0.0310 (12)	−0.0001 (9)	0.0039 (9)	−0.0013 (9)
C11	0.0215 (10)	0.0213 (11)	0.0182 (10)	−0.0021 (8)	0.0046 (8)	−0.0014 (8)
C12	0.0204 (10)	0.0246 (12)	0.0253 (11)	−0.0011 (9)	0.0016 (8)	0.0021 (9)
C13	0.0304 (12)	0.0229 (12)	0.0284 (12)	0.0034 (9)	0.0058 (9)	0.0046 (9)
C14	0.0380 (13)	0.0228 (12)	0.0274 (12)	−0.0071 (10)	0.0062 (10)	−0.0034 (9)
C15	0.0304 (12)	0.0308 (13)	0.0288 (12)	−0.0080 (10)	−0.0026 (10)	−0.0057 (10)
C16	0.0243 (11)	0.0274 (12)	0.0227 (11)	0.0000 (9)	0.0012 (9)	−0.0002 (9)
C17	0.0244 (11)	0.0183 (11)	0.0318 (12)	0.0071 (9)	−0.0049 (9)	−0.0025 (9)
C18	0.0348 (13)	0.0491 (16)	0.0288 (13)	0.0103 (12)	−0.0022 (10)	−0.0075 (11)
C19	0.060 (2)	0.083 (2)	0.0337 (15)	0.0341 (18)	−0.0099 (14)	−0.0188 (15)
C20	0.075 (2)	0.064 (2)	0.064 (2)	0.0447 (19)	−0.0437 (19)	−0.0425 (18)
C21	0.0466 (17)	0.0303 (15)	0.094 (3)	0.0147 (13)	−0.0409 (18)	−0.0256 (16)
C22	0.0279 (12)	0.0226 (13)	0.0642 (18)	0.0055 (10)	−0.0130 (12)	−0.0052 (12)
C23	0.0235 (10)	0.0174 (11)	0.0215 (11)	0.0031 (8)	0.0076 (8)	−0.0015 (8)
C24	0.0282 (12)	0.0214 (12)	0.0294 (12)	0.0034 (9)	0.0009 (9)	−0.0032 (9)
C25	0.0338 (13)	0.0218 (12)	0.0403 (14)	−0.0028 (10)	0.0104 (11)	−0.0096 (10)
C26	0.0528 (16)	0.0179 (12)	0.0360 (13)	0.0050 (11)	0.0173 (12)	0.0014 (10)
C27	0.0542 (16)	0.0285 (14)	0.0375 (14)	0.0123 (12)	−0.0063 (12)	0.0038 (11)
C28	0.0349 (13)	0.0259 (12)	0.0325 (13)	0.0035 (10)	−0.0055 (10)	0.0000 (10)
O1	0.0189 (7)	0.0204 (8)	0.0214 (7)	0.0014 (6)	0.0039 (6)	0.0043 (6)
P1	0.0184 (3)	0.0194 (3)	0.0232 (3)	0.0013 (2)	0.0013 (2)	0.0004 (2)
P2	0.0185 (3)	0.0207 (3)	0.0244 (3)	0.0002 (2)	0.0041 (2)	0.0021 (2)

### Geometric parameters (Å, º)

**Table:**

C1—C2	1.352 (3)	C14—H14	0.9500
C1—O1	1.381 (2)	C15—C16	1.386 (3)
C1—P1	1.808 (2)	C15—H15	0.9500
C2—C3	1.422 (3)	C16—H16	0.9500
C2—H2	0.9500	C17—C18	1.390 (3)
C3—C4	1.349 (3)	C17—C22	1.396 (3)
C3—H3	0.9500	C17—P2	1.839 (2)
C4—O1	1.386 (2)	C18—C19	1.381 (4)
C4—P2	1.812 (2)	C18—H18	0.9500
C5—C6	1.393 (3)	C19—C20	1.382 (5)
C5—C10	1.395 (3)	C19—H19	0.9500
C5—P1	1.838 (2)	C20—C21	1.373 (5)
C6—C7	1.388 (3)	C20—H20	0.9500
C6—H6	0.9500	C21—C22	1.387 (4)
C7—C8	1.379 (3)	C21—H21	0.9500
C7—H7	0.9500	C22—H22	0.9500
C8—C9	1.385 (3)	C23—C24	1.386 (3)
C8—H8	0.9500	C23—C28	1.390 (3)
C9—C10	1.389 (3)	C23—P2	1.830 (2)
C9—H9	0.9500	C24—C25	1.388 (3)
C10—H10	0.9500	C24—H24	0.9500
C11—C16	1.392 (3)	C25—C26	1.377 (4)
C11—C12	1.398 (3)	C25—H25	0.9500
C11—P1	1.835 (2)	C26—C27	1.384 (4)
C12—C13	1.380 (3)	C26—H26	0.9500
C12—H12	0.9500	C27—C28	1.375 (4)
C13—C14	1.385 (3)	C27—H27	0.9500
C13—H13	0.9500	C28—H28	0.9500
C14—C15	1.384 (3)		
			
C2—C1—O1	109.54 (17)	C15—C16—H16	119.7
C2—C1—P1	130.13 (16)	C11—C16—H16	119.7
O1—C1—P1	120.16 (14)	C18—C17—C22	118.7 (2)
C1—C2—C3	107.06 (18)	C18—C17—P2	124.21 (18)
C1—C2—H2	126.5	C22—C17—P2	117.14 (19)
C3—C2—H2	126.5	C19—C18—C17	120.6 (3)
C4—C3—C2	107.29 (18)	C19—C18—H18	119.7
C4—C3—H3	126.4	C17—C18—H18	119.7
C2—C3—H3	126.4	C18—C19—C20	120.0 (3)
C3—C4—O1	109.38 (17)	C18—C19—H19	120.0
C3—C4—P2	130.27 (16)	C20—C19—H19	120.0
O1—C4—P2	120.34 (14)	C21—C20—C19	120.2 (3)
C6—C5—C10	118.57 (19)	C21—C20—H20	119.9
C6—C5—P1	119.10 (16)	C19—C20—H20	119.9
C10—C5—P1	122.02 (16)	C20—C21—C22	120.0 (3)
C7—C6—C5	120.7 (2)	C20—C21—H21	120.0
C7—C6—H6	119.6	C22—C21—H21	120.0
C5—C6—H6	119.6	C21—C22—C17	120.4 (3)
C8—C7—C6	120.3 (2)	C21—C22—H22	119.8
C8—C7—H7	119.9	C17—C22—H22	119.8
C6—C7—H7	119.9	C24—C23—C28	118.4 (2)
C7—C8—C9	119.7 (2)	C24—C23—P2	123.17 (16)
C7—C8—H8	120.2	C28—C23—P2	118.26 (17)
C9—C8—H8	120.2	C23—C24—C25	120.5 (2)
C8—C9—C10	120.3 (2)	C23—C24—H24	119.8
C8—C9—H9	119.8	C25—C24—H24	119.8
C10—C9—H9	119.8	C26—C25—C24	120.5 (2)
C9—C10—C5	120.4 (2)	C26—C25—H25	119.8
C9—C10—H10	119.8	C24—C25—H25	119.8
C5—C10—H10	119.8	C25—C26—C27	119.3 (2)
C16—C11—C12	118.9 (2)	C25—C26—H26	120.3
C16—C11—P1	118.21 (16)	C27—C26—H26	120.3
C12—C11—P1	122.94 (16)	C28—C27—C26	120.2 (2)
C13—C12—C11	120.2 (2)	C28—C27—H27	119.9
C13—C12—H12	119.9	C26—C27—H27	119.9
C11—C12—H12	119.9	C27—C28—C23	121.1 (2)
C12—C13—C14	120.6 (2)	C27—C28—H28	119.4
C12—C13—H13	119.7	C23—C28—H28	119.4
C14—C13—H13	119.7	C1—O1—C4	106.71 (15)
C15—C14—C13	119.7 (2)	C1—P1—C11	101.99 (9)
C15—C14—H14	120.1	C1—P1—C5	101.71 (9)
C13—C14—H14	120.1	C11—P1—C5	101.16 (9)
C14—C15—C16	120.0 (2)	C4—P2—C23	101.00 (9)
C14—C15—H15	120.0	C4—P2—C17	102.65 (9)
C16—C15—H15	120.0	C23—P2—C17	100.63 (10)
C15—C16—C11	120.6 (2)		
			
O1—C1—C2—C3	1.1 (2)	C25—C26—C27—C28	1.9 (4)
P1—C1—C2—C3	−173.96 (16)	C26—C27—C28—C23	−0.9 (4)
C1—C2—C3—C4	−1.2 (2)	C24—C23—C28—C27	−0.7 (3)
C2—C3—C4—O1	0.8 (2)	P2—C23—C28—C27	−175.58 (19)
C2—C3—C4—P2	−177.79 (16)	C2—C1—O1—C4	−0.7 (2)
C10—C5—C6—C7	0.1 (3)	P1—C1—O1—C4	175.01 (14)
P1—C5—C6—C7	−173.69 (16)	C3—C4—O1—C1	−0.1 (2)
C5—C6—C7—C8	0.2 (3)	P2—C4—O1—C1	178.63 (14)
C6—C7—C8—C9	−0.6 (3)	C2—C1—P1—C11	114.0 (2)
C7—C8—C9—C10	0.8 (3)	O1—C1—P1—C11	−60.70 (17)
C8—C9—C10—C5	−0.5 (3)	C2—C1—P1—C5	−141.8 (2)
C6—C5—C10—C9	0.1 (3)	O1—C1—P1—C5	43.55 (17)
P1—C5—C10—C9	173.68 (16)	C16—C11—P1—C1	−124.03 (16)
C16—C11—C12—C13	−0.2 (3)	C12—C11—P1—C1	56.25 (19)
P1—C11—C12—C13	179.52 (16)	C16—C11—P1—C5	131.29 (16)
C11—C12—C13—C14	−0.2 (3)	C12—C11—P1—C5	−48.43 (18)
C12—C13—C14—C15	0.5 (3)	C6—C5—P1—C1	−147.20 (17)
C13—C14—C15—C16	−0.4 (3)	C10—C5—P1—C1	39.21 (19)
C14—C15—C16—C11	−0.1 (3)	C6—C5—P1—C11	−42.30 (18)
C12—C11—C16—C15	0.4 (3)	C10—C5—P1—C11	144.11 (17)
P1—C11—C16—C15	−179.37 (17)	C3—C4—P2—C23	−132.8 (2)
C22—C17—C18—C19	0.6 (4)	O1—C4—P2—C23	48.70 (17)
P2—C17—C18—C19	−180.0 (2)	C3—C4—P2—C17	123.5 (2)
C17—C18—C19—C20	−0.5 (4)	O1—C4—P2—C17	−54.96 (17)
C18—C19—C20—C21	−0.1 (5)	C24—C23—P2—C4	30.32 (19)
C19—C20—C21—C22	0.5 (4)	C28—C23—P2—C4	−155.10 (17)
C20—C21—C22—C17	−0.5 (4)	C24—C23—P2—C17	135.60 (18)
C18—C17—C22—C21	−0.1 (3)	C28—C23—P2—C17	−49.82 (18)
P2—C17—C22—C21	−179.55 (19)	C18—C17—P2—C4	53.1 (2)
C28—C23—C24—C25	1.3 (3)	C22—C17—P2—C4	−127.44 (18)
P2—C23—C24—C25	175.88 (16)	C18—C17—P2—C23	−50.9 (2)
C23—C24—C25—C26	−0.3 (3)	C22—C17—P2—C23	128.61 (18)
C24—C25—C26—C27	−1.4 (3)		

### Hydrogen-bond geometry (Å, º)

Cg is the centroid of ring C17–C22.

**Table:**

*D*—H···*A*	*D*—H	H···*A*	*D*···*A*	*D*—H···*A*
C27—H27···*Cg*^i^	0.95	3.11	3.736 (3)	125

Symmetry code: (i) *x*, *y*−1, *z*.
