# Supplementary figures and images for: Crystal structure of 2,5-bis­(di­phenyl­phosphan­yl)furan
Source: Acta Crystallogr E Crystallogr Commun. 2015 Nov 11;71(Pt 12):o922–3. doi: 10.1107/S2056989015020964 (PMC4719882; doi:10.1107/S2056989015020964)

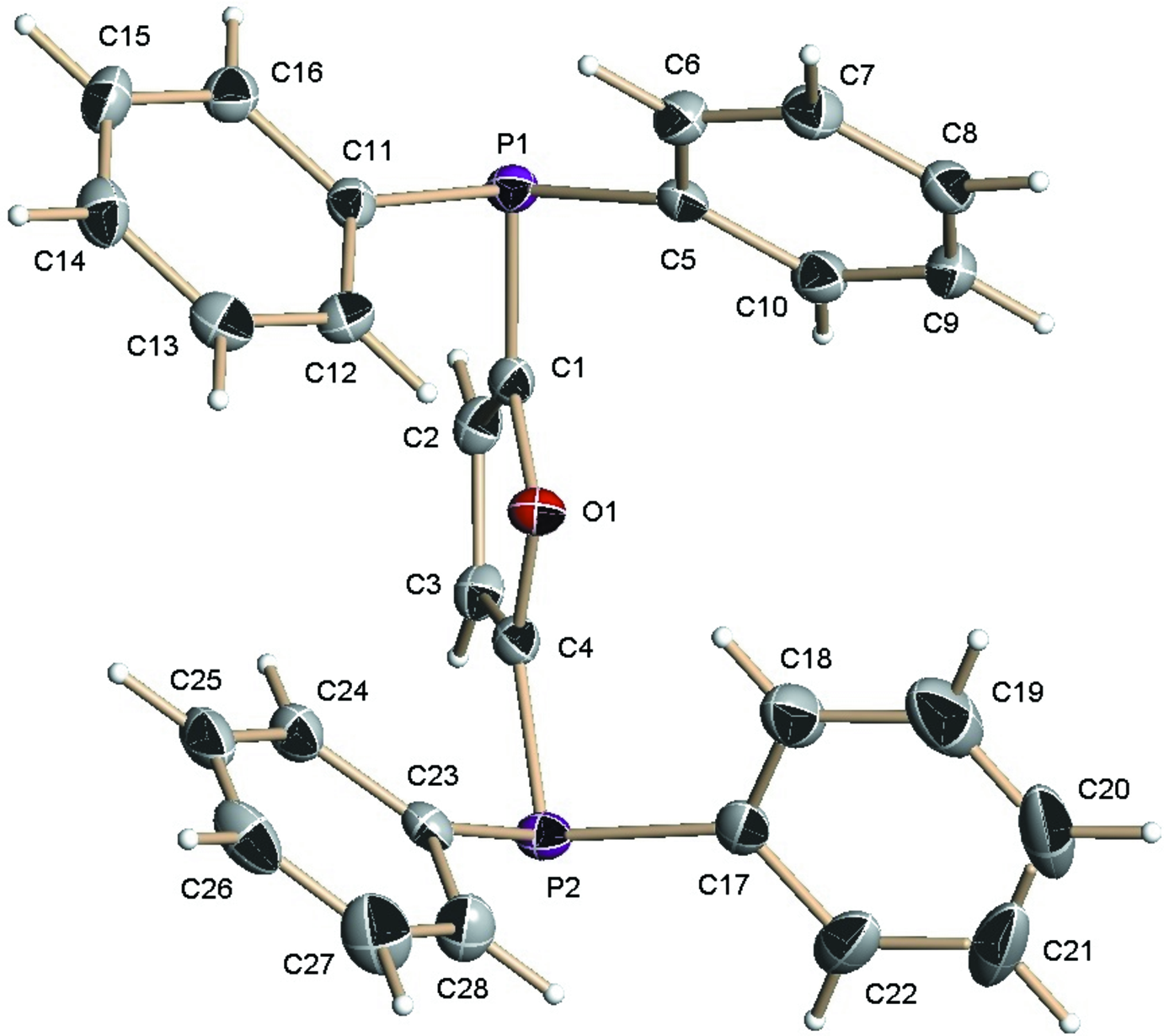

Supplement: Supplementary file 4 [file e-71-0o922-fig1.tif]

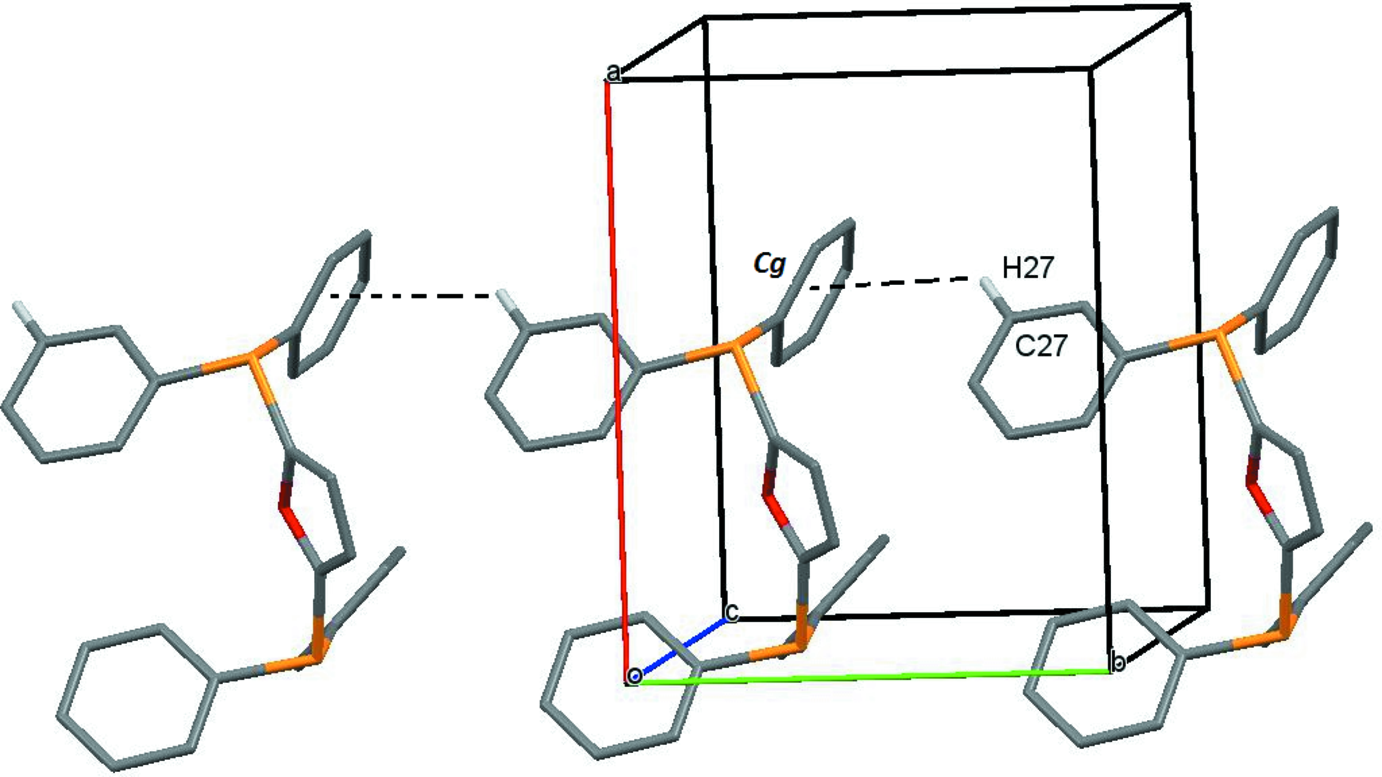

Supplement: Supplementary file 5 [file e-71-0o922-fig2.tif]
